# Demographic and Clinical Features and Prescribing Patterns of Psychotropic Medications in Patients with the Melancholic Subtype of Major Depressive Disorder in China

**DOI:** 10.1371/journal.pone.0039840

**Published:** 2012-06-29

**Authors:** Yu-Tao Xiang, Gang Wang, Chen Hu, Tong Guo, Gabor S. Ungvari, Amy M. Kilbourne, Kelly Y. C. Lai, Tian-Mei Si, Qi-Wen Zheng, Da-Fang Chen, Yi-Ru Fang, Zheng Lu, Hai-Chen Yang, Jian Hu, Zhi-Yu Chen, Yi Huang, Jing Sun, Xiao-Ping Wang, Hui-Chun Li, Jin-Bei Zhang, Helen F. K. Chiu

**Affiliations:** 1 Department of Psychiatry, Chinese University of Hong Kong, Hong Kong SAR, China; 2 Mood Disorders Center, Beijing Anding Hospital, Capital Medical University, Beijing, China; 3 University of Notre Dame Australia/Marian Centre, Perth, Western Australia, Australia; 4 Veterans Administration Ann Arbor Center for Clinical Management Research, Ann Arbor, Michigan, United States of America; 5 Department of Psychiatry, University of Michigan Medical School, Ann Arbor, Michigan, United States of America; 6 Key Laboratory of Mental Health, Ministry of Mental Health & Peking University Institute of Mental Health, Beijing, China; 7 Department of Epidemiology and Biostatistics, Peking University Health Science Center, Beijing, China; 8 Division of Mood Disorders, Shanghai Mental Health Center, Shanghai Jiao Tong University School of Medicine, Shanghai, China; 9 Shanghai Tongji Hospital, Tongji University Medical School, Shanghai, China; 10 Division of Mood Disorders, Shenzhen Mental Health Centre, Shenzhen, Guangdong Province, China; 11 The First Hospital of Harbin Medical University, Harbin, Heilongjiang Province, China; 12 Hangzhou Seventh People's Hospital, Hangzhou, Zhejiang Province, China; 13 West China Hospital, Sichuan University, Chengdu, Sichuan Province, China; 14 The Affiliated Brain Hospital, Nanjing Medical University, Nanjing, Jiangsu Province, China; 15 Mental Health Institute, The Second Xiangya Hospital, Central South University, Changsha, Hunan Province, China; 16 The Second Affiliated Hospital, College of Medicine, Zhejiang University, Hangzhou, Zhejiang Province, China; 17 The Third Affiliated Hospital of Sun Yat-Sen University, Guangzhou, Guangdong Province, China; Baylor College of Medicine, United States of America

## Abstract

**Background:**

Little has been known about the demographic and clinical features of the melancholic subtype of major depressive disorder (MDD) in Chinese patients. This study examined the frequency of melancholia in Chinese MDD patients and explored its demographic and clinical correlates and prescribing patterns of psychotropic drugs.

**Methods:**

A consecutively collected sample of 1,178 patients with MDD were examined in 13 psychiatric hospitals or psychiatric units of general hospitals in China nationwide. The cross-sectional data of patients’ demographic and clinical characteristics and prescriptions of psychotropic drugs were recorded using a standardized protocol and data collection procedure. The diagnosis of the melancholic subtype was established using the Mini International Neuropsychiatric Interview (MINI). Medications ascertained included antidepressants, mood stabilizers, antipsychotics and benzodiazepines.

**Results:**

Six hundred and twenty nine (53.4%) of the 1,178 patients fulfilled criteria for melancholia. In multiple logistic regression analyses, compared to non-melancholic counterparts, melancholic MDD patients were more likely to be male and receive benzodiazepines, had more frequent suicide ideations and attempts and seasonal depressive episodes, while they were less likely to be employed and receive antidepressants and had less family history of psychiatric disorders and lifetime depressive episodes.

**Conclusions:**

The demographic and clinical features of melancholic MDD in Chinese patients were not entirely consistent with those found in Western populations. Compared to non-melancholic MDD patients, melancholic patients presented with different demographic and clinical features, which have implications for treatment decisions.

## Introduction

Since the term melancholia was first operationally defined in the DSM-III [1], [2], melancholic depression, also described as “typical”, “endogenous”, “endogenomorphic”, “type A” and “autonomous” depression [3]–[5], has been the topic of continuous debate whether it represents an etiologically distinct syndrome or only a more severe variant of major depression. In DSM-III melancholia was not listed as a separate diagnosis [1]; in DSM-IV, it was still not separated from major depressive disorder (MDD) [6]. Both the DSM-III and DSM-IV melancholia criteria are inconsistent with features of melancholia described by empirical studies in which it is postulated as a distinct entity in terms of clinical profile, treatment response and biological markers [7], [8]. For example, compared to non-melancholic depression, melancholic depression usually respond better to electroconvulsive therapy (ECT) and broad-action tricyclic (TCA) than other classes of antidepressants [8]. Therefore, many researchers advocate that melancholia be listed as a distinct, identifiable and separate mood syndrome in the coming DSM-V classification [7].

In the past decade the characteristic features of melancholia have been comprehensively studied with varying results [9]–[11]. For example, some studies found that melancholia was more frequent in men [9], [12], which was not confirmed in other studies [11]. In addition, these findings were almost entirely obtained in Western populations. Given the fact that features of mood disorders are not independent of the complex interplay of the biopsychosocial environment [13], research findings in the West do not cover the full range of mood symptoms experienced by Chinese patients [14], [15]. Thus, it is necessary to examine the demographic and clinical features in Chinese melancholic patients.

There have been limited data on the frequency and features of melancholic MDD in East Asia including China. In a recent study Sun et al. [16] interviewed 1,970 Chinese women with MDD diagnosed by the Composite International Diagnostic Interview (CIDI) and found that in contrast to non-melancholia, melancholic MDD patients were older, had older age at onset, more episodes of illness and higher rates of lifetime generalized anxiety disorder. However, major weaknesses of this study including convenience sampling and inclusion of only female patients limit the generalization of the findings. In addition, the use of psychotropic drugs in melancholic MDD was not examined; this aspect has seldom been reported in the literature. We could not locate similar studies from other Eastern Asian countries, such as Japan and Korea, by searching English-language journals.

In order to improve the validity of diagnosis of bipolar disorders (BD) and rationalize its treatment, the Chinese Society of Psychiatry initiated a nation-wide study entitled the Diagnostic Assessment Service for People with Bipolar Disorders in China (DASP) that aims to test the usefulness of screening tools for BD in patients initially diagnosed as MDD [17], [18]. The current study, a secondary analysis of the data of the DASP project, set out (1) to determine the frequency of melancholia in Chinese patients with MDD based on the Mini International Neuropsychiatric Interview (MINI); and (2) to explore its demographic and clinical correlates and prescribing patterns of antidepressants, mood stabilizers, antipsychotics and benzodiazepines.

## Methods

### Ethics Statement

The study protocol was approved centrally by the Clinical Research Ethics Committee of Beijing Anding Hospital, Capital Medical University, China and the Clinical Research Ethics Committees of the respective study centers. All patients provided informed consent to participate in this study.

### Study Participants and Settings

The first survey of the DASP project was conducted in 13 major psychiatric hospitals/units located in north, south, east, west and central parts of China representing a range of clinical settings from September 1, 2010 through February 28, 2011. Patients were screened and enrolled if they were aged between 16 and 65 years, were inpatients or outpatients, had a diagnosis of DSM-IV MDD ascertained by the MINI, understood the aims of the study and provided informed consent. Exclusion criteria included (1) past diagnosis of BD; (2) history or ongoing significant medical or neurological condition(s); (3) depressive disorders secondary to a general medical or neurological condition; and (4) Having received electroconvulsive therapy (ECT) in the past month.

### Instrument and Assessment Procedure

Consecutive sampling method was used in this study. Patients with a diagnosis of MDD receiving treatment in the participating hospitals/units were consecutively referred by their treating psychiatrists to the research team to be screened for eligibility. Patients fulfilling the above entry criteria were invited to participate in the study.

The patients’ basic demographic and clinical data were collected with a questionnaire designed for the study in the course of a clinical interview supplemented by a review of their medical records. Following an earlier study [19], the diagnostic assessment of melancholia was conducted with the Chinese version of the MINI, Version 5.0, to establish DSM-IV diagnosis of the melancholic subtype of MDD [20], [21].

Prior to the study all thirteen raters were trained in the use of MINI in twenty patients with MDD. In this reliability exercise, the kappa values of their judgments on melancholia were more than 0.80 for each rater. After providing written consent following a full explanation about the study, participating patients met a rater for a confirmatory diagnostic interview.

## Analysis

Data were analyzed with the SPSS 13.0. Comparisons of the demographic and clinical characteristics and prescribing patterns of psychotropic drugs between melancholic patients and those without it were performed by multiple logistic regression analyses with the “Enter” method. The diagnosis of melancholia was entered as the dependent variable and demographic and clinical characteristics and prescribing patterns of psychotropic medications (see Table 1) were entered as independent variables. The level of significance was set at 0.05 (two-tailed).

**Table 1:** Basic demographic and clinical characteristics of MDD patients with or without melancholia.

	The whole sample(n = 1,178)		Nonmelancholic(n = 549)		Melancholic(n = 629)		Statistics a		
							O.R.	95% CI	P
	N	%	N	%	N	%			
Male	385	32.7	154	28.1	231	36.7	1.6	1.3, 2.1	<0.001
Married/cohabitating	824	69.9	379	69.0	445	70.7	1.1	0.8, 1.5	0.5
Employed	812	68.9	406	74.0	406	64.5	0.7	0.5, 0.9	0.004
Education									
Primary and junior secondary school	356	30.2	150	27.3	206	32.8	1.0	–-	–-
Senior secondary school	312	26.5	137	25.0	175	27.8	1.0	0.7, 1.4	0.8
College and university	465	39.5	241	43.9	224	35.6	0.8	0.5, 1.1	0.1
Postgraduate	45	3.8	21	3.8	24	3.8	1.0	0.5, 1.9	0.9
Depressive episodes with increased appetite and sleep and weight gain	179	15.2	90	16.4	89	14.1	0.9	0.6, 1.2	0.4
Depressive episodes with suicidal ideation and/or attempts	665	56.5	304	55.4	361	57.4	1.3	1.004, 1.6	0.047
Depressive episodes with psychotic symptoms	158	13.4	67	12.2	91	14.5	1.3	0.9, 1.9	0.1
Seasonal depressive episodes	134	11.4	56	10.2	78	12.4	1.7	1.2, 2.6	0.008
Family history of psychiatric disorders	203	17.2	114	20.8	89	14.1	0.6	0.5, 0.9	0.004
Any use of antidepressants	820	69.9	412	75.0	408	64.9	0.6	0.5, 0.8	<0.001
Any use of antipsychotics	226	19.2	104	18.9	122	19.4	1.1	0.8, 1.5	0.4
Any use of mood stabilizers	44	3.7	28	5.1	16	2.5	0.6	0.3, 1.1	0.1
Any use of benzodiazepines	192	16.3	77	14.0	115	18.3	1.6	1.1, 2.2	0.01
	**Mean**	**SD**	**Mean**	**SD**	**Mean**	**SD**			
Age (years)	40.5	12.8	40.29	12.6	40.8	13.1	1.0	0.9, 1.01	0.6
Age at onset (years)	34.6	12.5	33.9	11.9	35.3	13.0	1.0	0.9, 1.03	0.2
Lifetime depressive episodes	1.9	2.6	2.2	2.9	1.6	2.2	0.9	0.87, 0.98	0.005

a multiple logistic regression analyses with the nonmelancholic cohort as the reference group.

## Results

Altogether, 1,757 patients were invited to participate in the study; 270 (15.4%) refused, 309 (17.6%) fulfilled DSM-IV criteria for BD and 1,178 (67.0%) for MDD. In the MDD group, 629 (53.4%) had melancholia based on the MINI. Table 1 shows the basic demographic and clinical characteristics and the use of psychotropic drugs for the whole sample and separately by diagnoses. In multiple logistic regression analysis melancholic patients were more likely to be male, receive benzodiazepines and have more frequent suicide ideations and attempts and seasonal depressive episodes, while they were less likely to be employed and receive antidepressants, and had less family history of psychiatric disorders and lifetime depressive episodes.

## Discussion

The frequency of melancholia among MMD patients (53.4%) found in this study was within the range of the 20% to 80% reported in the literature [3]. Our result is similar to the findings in MDD patients from Western countries; for example 50% in a US community sample [22] and 44% in New Zealand outpatients [10], but considerably lower than a recent finding in China [16]. In a convenience sample of 1,970 female Chinese MDD patients 81.3% were diagnosed as melancholia based on the CIDI [16]. The wide discrepancy between Sun et al.’s finding and ours might be due to selection bias (convenience vs. consecutive sampling; female sex vs. both sexes) and interviewing techniques (the CIDI vs. MINI).

Sex difference in depression has been well reported with higher heritability of depression in women [23], [24]. However, it is unclear whether sex differences exist in frequency of melancholic depression. In line with some [9], [12], but not all [11] studies, the proportion of melancholia was more frequent in men than women in this study. Melancholic depression is characterized with cognitive slowing [6] and low level of functioning [11], which could perhaps account for the lower employment rate compared to non-melancholic MDD.

Melancholic patients had greater risk of suicidal ideation and attempts and more use of benzodiazepines in this study, which might be due to the severity of depression and more frequent comorbidity, such as anxiety, in this patient group [9]–[11]. Unlike previous findings in Western settings that melancholic patients have a larger number of episodes and increased familial loading [16], [22], in this study melancholia was associated with less family history of psychiatric disorders and lifetime depressive episodes.

Melancholia is usually more severe than the non-melancholic subtype [11], therefore it is reasonable to assume that they are more likely to receive antidepressant treatment. In this study, surprisingly only 64.9% of melancholic patients were on antidepressants, lower than the figure (75.0%) in non-melancholic patients. We cannot explain this striking finding based on the current study design; this result needs to be replicated in future surveys.

Melancholic patients had more seasonal episodes in this study, a finding that has not been reported previously. We also have no clear explanation for this pattern except assuming that seasonality might be a manifestation of a disturbed biological rhythm and, as such, a sign of the biological nature of melancholic depression.

The merits of this study are its large, multicentre sample and the standardized diagnostic assessment of melancholic MDD. However, the results should be interpreted with caution due to certain limitations. First, the cross-sectional nature of the study could not reflect the causal relationships between demographic and clinical variables and melancholic MDD. Second, no standardized instruments were used to measure the severity of depressive symptoms. Third, some potentially important clinical and psychosocial factors, such as history of childhood abuse, length of depressive episodes and rating of stressful life events, were not measured. Fourth, the sampling was not random, thus the sample cannot adequately represent all MDD patients in China. However, the participating centres are major tertiary institutions located in different parts of China, hence the current sample is broadly representative of the real life clinical practice in China. Finally, most background information was gleaned from patients or their medical records that is a hardly reliable source.

In conclusion, there are significant differences between non- and melancholic MDD in a host of demographic and clinical characteristics and antidepressant prescription patterns that have implications for treatment decisions. In addition, although it has been reported that melancholic MDD is a biologically more distinct syndrome in a number of studies, its demographic and clinical features in Chinese patients, such as less lifetime depressive episodes, less likely to receive antidepressants and more seasonal episodes, are neither entirely consistent with those found in Western melancholic patients, nor have been reported previously.
